# Fixel-based analysis reveals detailed white matter changes in semantic dementia

**DOI:** 10.1007/s00429-025-03064-7

**Published:** 2026-01-07

**Authors:** Maria Luisa Mandelli, Yann Cobigo, Ilaria Perretti, Dana Leichter, Celina Alba, Rian Bogley, Nick Wellman, Siddarth Ramkrishnan, Zachary A. Miller, Bruce L. Miller, William W. Seeley, Howard J. Rosen, Maria Luisa Gorno-Tempini

**Affiliations:** 1 Memory and Aging Center, Department of Neurology, University of California San Francisco, 675 Nelson Rising Lane, Suite 190, San Francisco, CA 94158 USA; 2 Department of Brain and Behavioral Sciences, University of Pavia, Pavia, Italy

**Keywords:** Fixel-based analysis, Diffusion brain imaging, Tract-based spatial statistics, White matter changes, Semantic dementia, Anterior temporal lobe, Anterior commissure

## Abstract

Accurately characterizing white matter (WM) microstructure is critical for understanding neurodegenerative diseases such as semantic dementia (SD). Regionally constrained techniques like tract-based spatial statistics (TBSS) rely on diffusion-tensor imaging (DTI) and assume a single fiber population per voxel, limiting their sensitivity to complex architecture. Fixel-based analysis (FBA) overcomes these constraints by resolving multiple fiber populations (fixels) within a single voxel, enabling more anatomically specific assessment of WM organization. Multi-shell diffusion MRI from 16 semantic-variant PPA (svPPA) and 15 semantic-behavioral fronto-temporal dementia (sbvFTD) cases—with imaging-confirmed left- and right-predominant temporal atrophy, respectively—and 44 neurologically healthy controls were analyzed using both TBSS-DTI and whole-brain FBA. Fiber-specific metrics of fiber density and cross-section were evaluated alongside conventional DTI measures. Both methods confirmed damage to the anterior temporal lobe (ATL) connected tracts—the uncinate fasciculus, inferior longitudinal fasciculus, inferior fronto-occipital fasciculus, and temporal projections of the arcuate fasciculus. FBA further detected involvement of juxtacortical and other pathways that were not captured by TBSS, including the tapetum and anterior commissure, projections to the parahippocampal gyrus and amygdala, and longer-range parietal connections. By capturing multiple fiber populations within each voxel, FBA provides greater anatomical precision and sensitivity to micro- and macro-structural changes than TBSS. This fiber-specific framework enables a more comprehensive mapping of WM degeneration in SD and helps delineate early alterations in commissural and mesial-temporal pathways that may underlie disease spread and cognitive decline.

**Supplementary Information:**

The online version contains supplementary material available at 10.1007/s00429-025-03064-7.

## Introduction

Over the past two decades, diffusion MRI (dMRI) has significantly advanced our ability to study white matter (WM) microstructure, enabling non-invasive, in-vivo visualization of fiber pathways and their organization. By quantifying WM integrity, dMRI provides critical insights into neurodegenerative disease progression, revealing subtle microstructural alterations often undetectable by conventional MRI (Goveas et al. 2015). These findings underscore the role of WM connectivity disruptions in conditions such as Alzheimer’s disease, mild cognitive impairment, amyotrophic lateral sclerosis (Li et al. 2012; Oishi et al. 2011), and frontotemporal dementia (Agosta et al. 2010; Galantucci et al. 2011; Whitwell et al. 2010), supporting the conceptualization of neurodegenerative disorders as network-level disconnection syndromes (Agosta et al. 2012; Fletcher and Warren 2011). However, conventional dMRI methods, such as diffusion tensor imaging (DTI), are limited in capturing the complexity of WM fiber geometry, reducing biological specificity and challenging interpretation (Basser et al. 1994; Jeurissen et al. 2019; Pierpaoli et al. 2001; Wheeler-Kingshott and Cercignani 2009). Metrics like fractional anisotropy (FA) and mean diffusivity (MD), typically analyzed using voxel-based approaches or tract-based spatial statistics (TBSS) (Smith et al. 2006), average diffusion properties within each voxel, obscuring the presence of multiple fiber populations and their unique characteristics. To overcome these limitations, Fixel-based analysis (FBA) has been introduced as an anatomically precise framework for studying WM alterations (Raffelt et al. 2015, 2017). Unlike conventional approaches, FBA differentiates distinct fiber populations (‘fixels’) within each voxel, allowing fiber-specific quantification of micro- and macrostructural changes. Furthermore, FBA facilitates a whole-brain approach without restricting measurements to the white matter “skeleton,” as required by TBSS. This approach is particularly well-suited for detecting subtle changes in juxtacortical regions and capturing distributed, network-level processes underlying neurodegenerative diseases.

Semantic-variant primary progressive aphasia (svPPA) (Gorno-Tempini et al. 2011), also known as semantic dementia (SD) (Hodges et al. 1992; Snowden et al. 1989), is a clinical syndrome within the frontotemporal dementia spectrum characterized by focal, often asymmetric, atrophy of the anterior temporal lobes (ATLs). Degenerative changes typically involve the temporal poles, medial temporal limbic structures (including the hippocampi and amygdalae), anterior lateral temporal neocortical areas, and posterior insulae (Binney et al. 2016; Bocchetta et al. 2020; Borghesani et al. 2020; Chan et al. 2001; Ding et al. 2016). These regions form broader cortical and subcortical networks supporting semantic memory, and socio-emotional processing (Benhamou et al. 2020; Fletcher and Warren 2011; Guo et al. 2013; Seeley 2008), highlighting the importance of investigating connectivity within these neural circuits.

FBA offers a robust framework for assessing structural connectivity in SD by analyzing distinct fiber populations within each voxel. This enhanced specificity enables detection of subtle alterations in small, intricate pathways—such as the anterior commissure—that conventional DTI might overlook. By accurately capturing complex neuroanatomical configurations, FBA provides deeper insights into network-level disruptions associated with clinical manifestations of SD. Although svPPA (and semantic dementia) has historically been associated with left-predominant ATL atrophy (Chan et al. 2001; Hodges et al. 1992; Mummery et al. 2000; Snowden et al. 1989), recent studies suggest that at least one-third of cases show right-predominant ATL degeneration (Borghesani et al. 2020; Chan et al. 2009; Edwards-Lee et al. 1997; Gainotti et al. 2003; Hodges et al. 2004; Kumfor et al. 2016; Patterson et al. 2007; Snowden et al. 2018). These cases have recently been classified as semantic behavioral variant frontotemporal dementia (sbvFTD) (Younes et al. 2022), or right temporal FTD. Here, we use “semantic dementia” as an umbrella term encompassing both svPPA and sbvFTD. Regardless of initial lateralization, the disease eventually progresses contralaterally and extends to orbitofrontal, anterior cingulate, and temporoparietal regions (Borghesani et al. 2020; Brambati et al. 2009; Josephs et al. 2011; Kumfor et al. 2016; Rohrer et al. 2009; Spinelli et al. 2017). Investigating both left- and right-sided pathways is therefore crucial for capturing the full disease spectrum.

DTI studies on SD have consistently highlighted involvement of major WM tracts connecting the ATL to other cortical regions, including the inferior longitudinal, arcuate, and uncinate fasciculi, while showing relative sparing of dorsal frontoparietal pathways (Acosta-Cabronero et al. 2011; Agosta et al. 2010; Whitwell et al. 2010). However, interhemispheric connections—such as the anterior commissure or the tapetum of the corpus callosum (CC)—have received less attention, likely due to their small size and complex configuration, complicating delineation with conventional DTI approaches. Detailed examination of these pathways could significantly enhance understanding of WM microstructural alterations across hemispheres and provide new insights into network-driven degeneration underlying SD.

In this study, we investigated WM integrity in SD using both FBA and TBSS-DTI applied to high-angular-resolution, multi-shell MR diffusion images. Our sample included 16 individuals with svPPA, 15 individuals with sbvFTD, and a control group of 44 healthy control participants. We hypothesized that the fiber-specific modeling of FBA would reveal additional or more anatomically detailed alterations within intra- and interhemispheric pathways connecting the ATL and related regions, relative to conventional DTI approaches, thereby improving our understanding of the tract-specific WM disruptions underlying SD.

## Methods

### Participants

Patients with a clinical diagnosis of svPPA or sbvFTD were recruited from the database available at the Memory and Aging Center, University of California, San Francisco (MAC-UCSF), according to previously published diagnostic criteria (Gorno-Tempini et al. 2011; Younes et al. 2022). To focus the analysis on the earlier phase of the disease, patients were included if their Mini-Mental State Examination (MMSE) was greater than or equal to 14, and their Clinical Dementia Rating (CDR) was less than or equal to 2 at the time of imaging. Participants were included only if multi-shell diffusion-weighted imaging were available, as this acquisition enables advanced fiber-orientation modelling required for FBA and improved tract-specific estimation (Jeurissen et al. 2014). Patients were classified as having right- or left- predominant ATL atrophy based on a lateralization index derived from structural brain MRI, as detailed below. Accordingly, cases showing prominent bilateral temporal atrophy or atrophy extending into the frontal lobes were excluded from the analysis. Neurologically healthy, community-dwelling older adults were recruited from the BRain Aging Network for Cognitive Health (BRANCH) study at the MAC-UCSF, which includes in-person behavioral and neuroimaging assessments. Controls participants were verified as neurologically healthy through multidisciplinary evaluation, including neurological examination, neuropsychological testing, and cognitive assessment (Kramer et al. 2003).

### Brain MRI acquisition

MRI scans were collected at the UCSF Neuroscience Imaging Center on a 3T Siemens Magnetom Prisma Scanner. Magnetization-prepared rapid gradient-echo (MPRAGE) sequences were used to acquire T1-weighted images with the following parameters: 160 sagittal slices, isotropic voxel size = 1mm^3^, repetition time (TR)/echo time (TE) = 2300/2.98 ms, inversion time = 900 ms, flip angle = 9°, field of view (FoV) read of 256 × 256 mm^2^, and an integrated parallel acquisition technique (iPAT) acceleration factor of 2. Diffusion-weighted images were collected with a single-shot spin-echo echo-planar imaging (SE-EPI) sequence with the following parameters: 69 contiguous axial slices with in-plane resolution = 2 × 2mm^2^, TR/TE = 2420/72.20 ms, flip angle = 85°, FoV of 220 × 220 mm^2^, 96 non-collinear diffusion sensitization directions at b = 2500 s/mm^2^, 48 directions at b = 1000 s/mm^2^, 30 directions at b = 500 s/mm^2^, and 10 volumes at b = 0, with an iPAT acceleration factor of 2 and multiband acceleration factor of 3. Two additional b = 0 volumes were acquired with opposite phase-encoding directions (anterior/posterior and posterior/anterior) for distortion correction. Diffusion-encoding gradients were distributed over a sphere and acquired using a monopolar scheme. Visual quality inspections were conducted for all MRI data, and any scans showing anomalies, artifacts, excessive motion, or significant white matter hyperintensity were excluded.

### Structural brain processing

#### Temporal atrophy index for group assignment

This study includes participants with moderate and focal atrophy predominantly affecting either the left or right temporal regions. We calculated an atrophy index reflecting the lateralization of temporal atrophy and its proportion relative to frontal lobe involvement, following procedures previously described (Younes et al. 2022). We extracted the cortical volume within the temporal and frontal lobes using the Desikan atlas as a reference (Desikan et al. 2006) after processing the structural images (Ashburner and Friston 2005, 2011; Manjón et al. 2010; Rajapakse et al. 1997) through the Computational Anatomy Toolbox within the framework of Statistical Parametric Mapping software (SPM12; fil.ion.ucl.ac.uk/spm/software/spm12). Specifically, the temporal regions consisted of the temporal pole, entorhinal cortex, fusiform gyrus, banks of the superior temporal sulcus, transverse temporal gyrus, and inferior, middle, and superior temporal gyri. The frontal brain regions included the inferior, middle, and superior frontal gyri. For each anatomical region, volume W-scores were generated from normative data derived from a control group of healthy individuals, adjusted for age and sex. W-scores are standardized with a mean of 0 and a standard deviation of 1; scores of + 1.65 and − 1.65 denote the 95th and 5th percentiles, respectively, indicating cortical volumes that are significantly larger or smaller compared to the normative cohort. Patients were considered to be in the left ATL-predominant atrophy group if the highest W-score occurred in the left temporal region and the ratio of the mean frontal to the mean left temporal W-score was less than 0.50. Similarly, patients were included in the right ATL-predominant atrophy group if the highest W-score occurred in the right temporal regions and the ratio of the mean frontal to mean right temporal W-score was less than 0.50. This stratification ensures the selection of patients who have relatively intact frontal lobes, thus likely representing those who are at an earlier stage of the disease from an anatomical perspective.

### Diffusion-weighted imaging analysis

#### Diffusion preprocessing

Diffusion-weighted images were first denoised (Veraart et al. 2016) and corrected for Gibbs ringing artifacts (Kellner et al. 2016), followed by motion, eddy-current, and susceptibility distortion correction using the FMRIB Software Library (FSL) (Smith et al. 2004). Susceptibility-induced off-resonance fields were estimated from two volumes acquired with opposite phase-encoding directions using the FSL ‘topup’ tool, and eddy-current distortions and head motion were corrected using the FSL ‘eddy’ tool (Andersson et al. 2003; Andersson and Sotiropoulos 2016).

#### Diffusion tensor imaging and TBSS

Tensor-based registration was used to generate single-subject tensor maps aligned to a common template (Keihaninejad et al. 2012; Zhang et al. 2007). An inter-subject group template was generated through iterative linear and non-linear registrations of the diffusion tensor images. Four diffusion metrics - FA, MD, radial diffusivity (RD), and axial diffusivity (AD) - were derived from the fitted tensors for each subject. Visual quality inspection was conducted to check for any registration errors. Analysis of the DTI metrics was performed using tract-based spatial statistics (TBSS). The FA maps for each subject were averaged to generate a single mean FA map, which was thinned with a threshold of 0.2 to create a skeletonized FA image representing the center of the WM tracts common to all participants (Smith et al. 2006). The aligned FA, MD, RD, and AD maps for each subject were projected onto this FA skeleton. The resulting skeletonized maps were used in voxel-wise cross-subject statistical analysis, using FSL *randomise* with 5000 permutations (Winkler et al. 2014). The two cohorts of patients (svPPA and sbvFTD) were compared respectively to the healthy control group. All models were controlled for the potential confounding effects of age, sex, and total intracranial volume (TIV). P-values were corrected with threshold-free cluster enhancement, and *P* < 0.05 was set as the significance threshold (Smith and Nichols 2009). Anatomical localization of the significant results along the main tracts was performed using the HCP1065 Population-Averaged Tractography Atlas (Yeh 2022). The Supplementary Material presents a color-coded map of the white-matter tracts described in this study; the same color scheme and section references are used in Figs. 2 and 3.

### Fixel-based morphometry

Fixel-based morphometry was performed using the MRtrix3 toolbox (Raffelt et al. 2017; Tournier et al. 2019). The preprocessed diffusion images were upsampled to an isotropic resolution of 1.25 mm to improve downstream spatial alignment and fixel segmentation. A multi-shell, multi-tissue constrained spherical deconvolution (MSMT-CSD) approach (Jeurissen et al. 2014; Tournier et al. 2004, 2007) was applied to model multiple tissue compartments within each voxel. Response functions for white matter (WM), gray matter (GM), and cerebrospinal fluid (CSF) were estimated using the *Dhollander* algorithm (Dhollander et al. 2021), and MSMT-CSD was then performed to compute the WM fiber orientation distributions (FODs) for each subject. Subsequently, joint bias-field correction and global intensity normalization were applied across subjects and tissue types (Raffelt et al. 2017). A study-specific FOD template was created using all participants, and individual subjects’ FODs were nonlinearly registered to this template. In template space, fixel segmentation was performed to extract distinct fiber populations (fixels) within each voxel, forming the basis for subsequent morphometric analyses.

#### FBA metrics

Fiber Density (FD) is a microstructural measure derived from the FOD. Within each voxel, the FOD is examined to identify its distinct lobes, each lobe representing a dominant fiber orientation or fixel. The amplitude of a given lobe is integrated along its direction, yielding FD, which approximates the intra-axonal volume fraction of that fiber population and is sensitive to axonal density, packing, or caliber, features related to axonal integrity and conduction capacity. Fiber cross-section (FC) is a macrostructural metric derived from the orthogonal scaling component of the deformation field; it quantifies how the cross-sectional area of a given fiber bundle must change to match the population template, capturing large-scale morphological alterations such as tract thinning or compression associated with atrophy. Their combined measure (FDC, fiber density and cross-section) was computed as the product of FD and FC (Dhollander et al. 2021), integrating information on both microscopic axonal content and macroscopic bundle morphology to provide a comprehensive index of white-matter integrity. Although these metrics are strongly linked to biological substrates of white matter, they remain indirect proxies of tissue microstructure and can be influenced by factors such as myelin loss, inflammation, or iron deposition.

#### Statistical analysis

Group comparisons of FD, FC, and FDC were performed at each fixel using a general linear model comparing (i) svPPA versus healthy controls and (ii) sbvFTD versus healthy controls, with sex and age included as covariates. Statistical significance was determined at a family-wise error (FWE)-corrected threshold of *p* < 0.05. Connectivity-based smoothing and statistical inference were performed using connectivity-based fixel enhancement with two million streamlines from the template tractogram and default smoothing parameters (10 mm full-width at half-maximum, C = 0.5, E = 2, H = 3) (Raffelt et al. 2015). FWE-corrected p-values were assigned to each fixel using non-parametric permutation testing with 5000 permutations (Nichols and Holmes 2002).

## Results

### MRI data quality control

The inclusion criteria required an MMSE score greater than or equal to 14, no motion or other MRI artifacts, and no evidence of white matter hyperintensity (Fazekas > 2). Of the 92 scans initially selected for this study (*n* = 23 svPPA, *n* = 23 sbvFTD, *n* = 44 healthy controls), several did not meet these criteria or failed the image quality control and were excluded from further processing and analysis. Seven of the 23 individuals with svPPA were excluded: six for MMSE scores below 14, and one due to atrophy extending into the left frontal region. Eight of the 23 individuals with sbvFTD were excluded: two for motion artifacts, one for extensive white matter hyperintensity and advanced age, two for atrophy extending into the frontal lobes, two for bilateral temporal atrophy, and one who showed no detectable atrophy and therefore did not meet imaging-supported diagnostic criteria. In total, 17 scans were excluded, leaving a final sample of 75 participants (16 svPPA, 15 sbvFTD, and 44 healthy controls). The included patient groups showed imaging findings consistent with the expected left- or right-lateralized temporal atrophy profiles. Demographic and clinical characteristics across groups are summarized in Table 1.

**Table 1:** Demographic and clinical characteristics of the study groups

	svPPA (*n* = 16)	sbvFTD (*n* = 15)
*Demographics*		
Age, mean (years)	67.1 ± 7.3	66.1 ± 6.9
Education, mean (years)	15.4 ± 3.3	15.8 ± 2.7
Sex, n female (%)	8 (50)	6 (40)
Handedness, n right (%)	15 (94)	12 (80)
*Disease severity*		
MMSE (30)	22.7 ± 5.3	24.5 ± 4.0
CDR Global (3)	0.7 ± 0.3	0.8 ± 0.5
CDR Sum of Boxes (18)	3.7 ± 2.3	4.3 ± 2.7
*Gray matter severity (W score)*		
Frontal atrophy	– 0.79 ± 0.98	– 1.12 ± 1.31
Left Temporal atrophy	– 6.22* ± 1.51	– 4.22 ± 1.56
Right Temporal atrophy	– 2.20 ± 1.87	– 6.23* ± 1.34
Ratio Frontal/Temporal Asymmetry	0.13 ± 0.16	0.15 ± 0.20

### Gray matter analysis

**Fig. 1:**
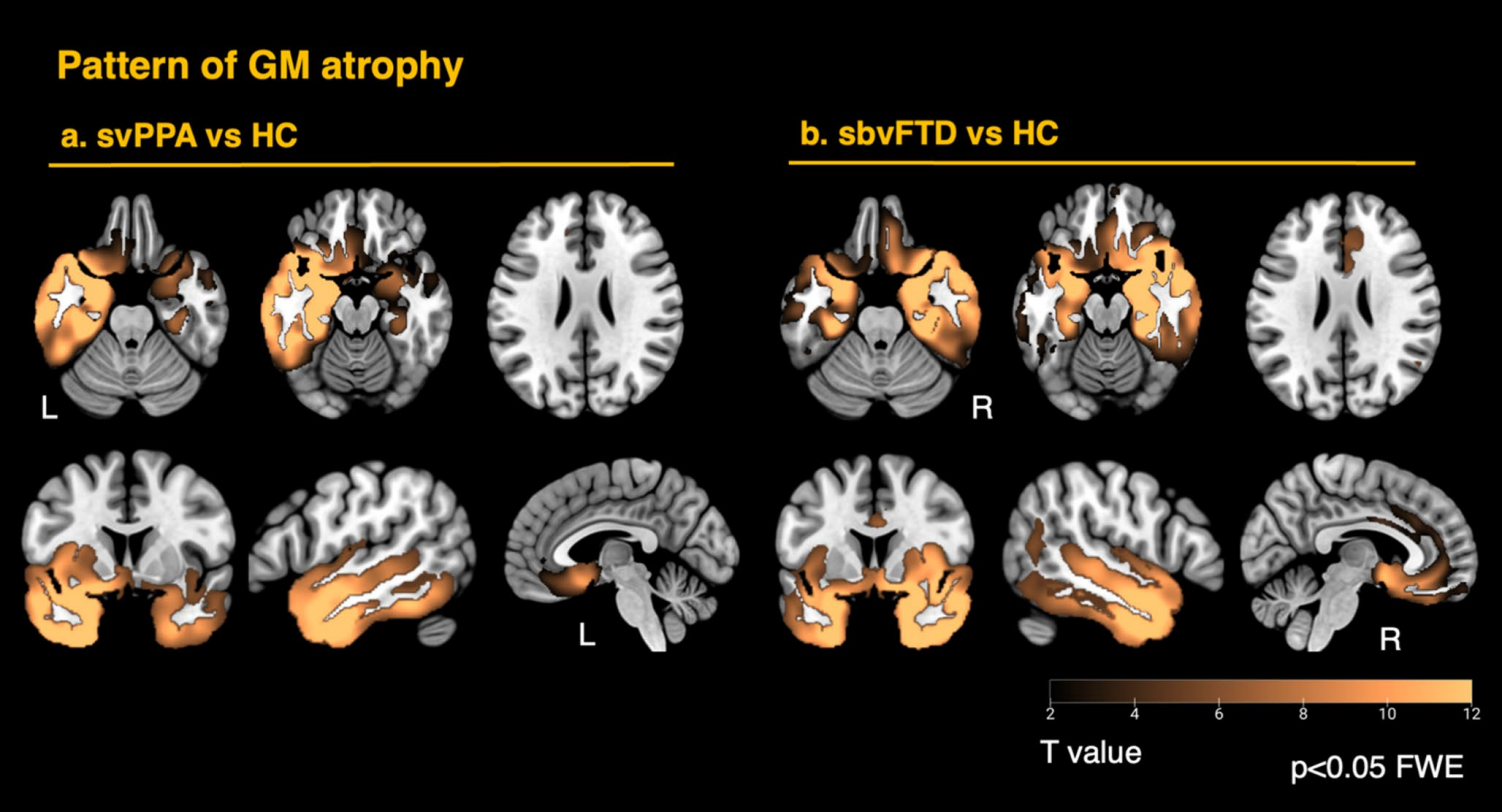
Gray-matter atrophy in the two anterior-temporal variants. Voxel-based morphometry maps illustrate convergent atrophy in the temporal pole, amygdala–hippocampal complex, and ventral limbic structures in both patient groups. **a** Semantic-variant primary progressive aphasia (svPPA) shows left-dominant loss; **b** semantic-behavioral variant frontotemporal dementia (sbvFTD) shows the right-lateralized mirror pattern with additional anterior cingulate cortex involvement. Warm colors indicate increasing *t*-values (greater atrophy relative to the healthy controls group). Statistical maps were family-wise error (FWE) corrected at *p* < 0.05)

Gray matter atrophy patterns confirmed predominant involvement of temporal and limbic regions, showing asymmetrical distributions between the two patient groups (Fig. 1). The overall atrophy patterns were largely mirrored between variants, although the sbvFTD group exhibited slightly greater gray matter loss, particularly in the anterior cingulate cortex. This difference may reflect the tendency for individuals with predominant right ATL involvement—who often present with behavioral symptoms - to seek clinical evaluation at a later stage than those with predominant left ATL involvement, who are typically diagnosed earlier due to language-related deficits.

### TBSS analysis of DTI metrics

#### Differences in SvPPA versus healthy controls

**Fig. 2:**
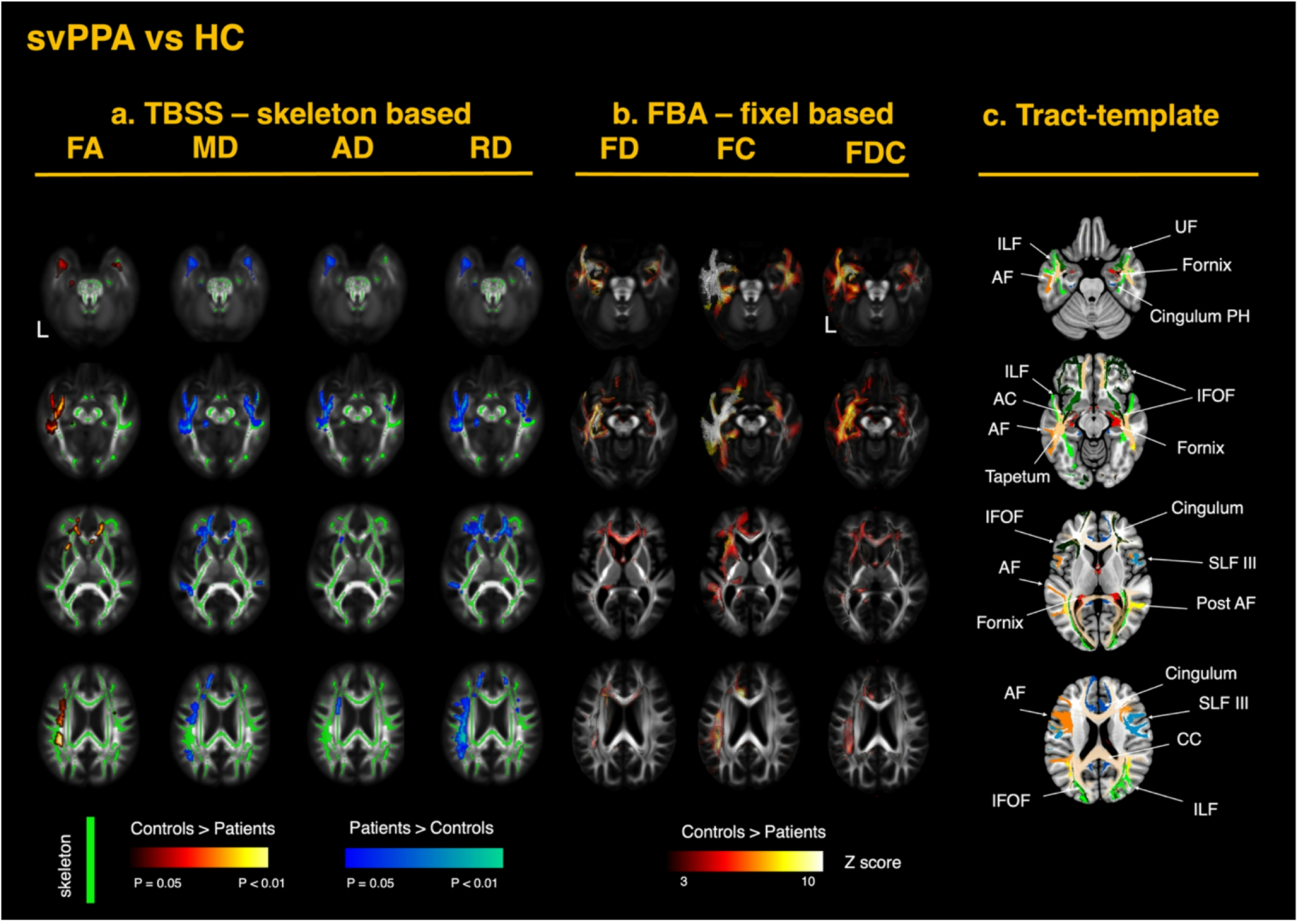
White matter alterations in semantic variant PPA (svPPA) relative to healthy controls. **a** TBSS-skeleton-based analysis: group contrasts of diffusion-tensor–derived metrics—fractional anisotropy (FA), mean diffusivity (MD), axial diffusivity (AD), and radial diffusivity (RD)—are shown on the TBSS skeleton (green) overlaid on the MNI-152 template. The color scale represents p-values after family-wise error (FWE) correction across the whole brain (*p* < 0.05). Warm colors indicate greater metric values in controls than in patients, whereas cool colors indicate the opposite pattern. **b** FBA—fixel-based analysis: corresponding contrasts for fiber density (FD), fiber cross-section (FC), and the combined metric fiber density and cross section (FDC). The color scale represents Z-scores for fixels that survived family-wise error (FWE) correction at *p* < 0.05. **c** Tracts template: axial slices highlight the principal tracts exhibiting significant change, including the uncinate fasciculus (UF), inferior longitudinal fasciculus (ILF), inferior fronto-occipital fasciculus (IFOF), arcuate fasciculus (AF), tapetum, anterior commissure (AC), fornix, parahippocampal cingulum, superior longitudinal fasciculus (SLF), posterior thalamic radiation, and corpus callosum (CC). Each row represents a different axial slice along the z-axis (MNI coordinates: z = 40, 54, 70 and 88 mm)

In the svPPA group, TBSS analysis revealed significant changes in the DTI metrics, with decreased FA and increased diffusivity metrics (MD, AD, RD) primarily in the supratentorial WM tracts connected to the left ATL, including the inferior longitudinal fasciculus (ILF), the uncinate fasciculus (UF) and lateral portions of the inferior fronto-occipital fasciculus (IFOF). Other WM structures also showed significant involvement, including the temporal and parietal projections of the left arcuate fasciculus (AF), the left external capsule, and key limbic system structures such as the fornix and left parahippocampal gyrus. Additionally, significant changes were noted in the genu of the CC and bilaterally in the anterior corona radiata. A summary of the TBSS findings for svPPA is displayed in Fig. 2A. Overall, FA showed the strongest hemispheric differences, whereas increased MD was more bilateral in the ventral tracts. RD was more sensitive than AD, primarily driving the observed MD effects. This pattern supports previous studies suggesting that RD is the most sensitive DTI metric for detecting pathological changes (Galantucci et al. 2011; Acosta-Cabronero et al. 2011; Agosta et al. 2012, 2015).

#### Differences in SbvFTD versus healthy controls

**Fig. 3:**
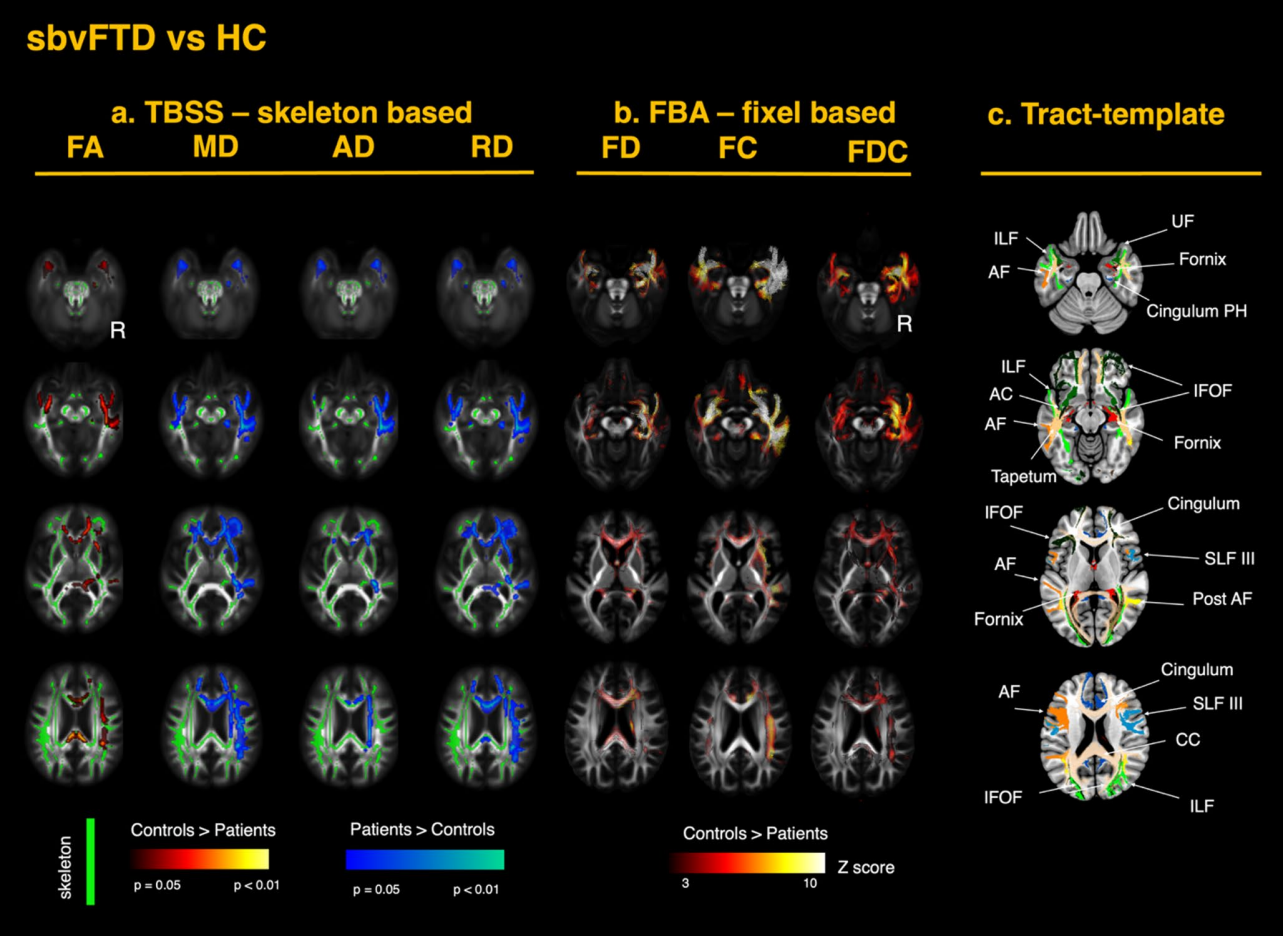
White matter alterations in semantic behavioral variant frontotemporal dementia (sbvFTD) relative to healthy controls. **a** TBSS-skeleton-based analysis: group contrasts of diffusion-tensor–derived metrics—fractional anisotropy (FA), mean diffusivity (MD), axial diffusivity (AD), and radial diffusivity (RD)—are shown on the TBSS skeleton (green) overlaid on the MNI-152 template. The color scale represents p-values after family-wise error (FWE) correction across the whole brain (*p* < 0.05). Warm colors indicate greater metric values in controls than in patients, whereas cool colors indicate the opposite pattern. **b** FBA—fixel-based analysis: corresponding contrasts for fiber density (FD), fiber cross-section (FC), and the combined metric fiber density-and-cross-section (FDC). The color scale represents Z-scores for fixels that survived family-wise error (FWE) correction at *p* < 0.05. **c** Tracts template: axial slices highlight the principal tracts exhibiting significant change, including the uncinate fasciculus (UF), inferior longitudinal fasciculus (ILF), inferior fronto-occipital fasciculus (IFOF), arcuate fasciculus (AF), tapetum, anterior commissure (AC), fornix, parahippocampal cingulum, superior longitudinal fasciculus (SLF), posterior thalamic radiation, and corpus callosum (CC). Each row represents a different axial slice along the z-axis (MNI coordinates: z = 40, 54, 70 and 88 mm)

A similar but mirrored pattern of changes was observed in the sbvFTD group, involving WM tracts connected to the right ATL and the limbic system, with additional involvement of the body and splenium of the CC, which were not observed in svPPA. This group exhibited more bilateral involvement overall, consistent with the gray matter atrophy pattern. A summary of the TBSS findings for sbvFTD is displayed in Fig. 3A, showing decreased FA and increased diffusivity metrics across tracts analogous to those affected in svPPA. As in svPPA, RD emerged as the most sensitive metric. The consistency of these findings across these two variants suggests a common pathophysiological mechanism underlying white-matter degeneration in semantic dementia.

### Fixel-based analysis

#### Differences in SvPPA versus healthy controls

**Fig. 4:**
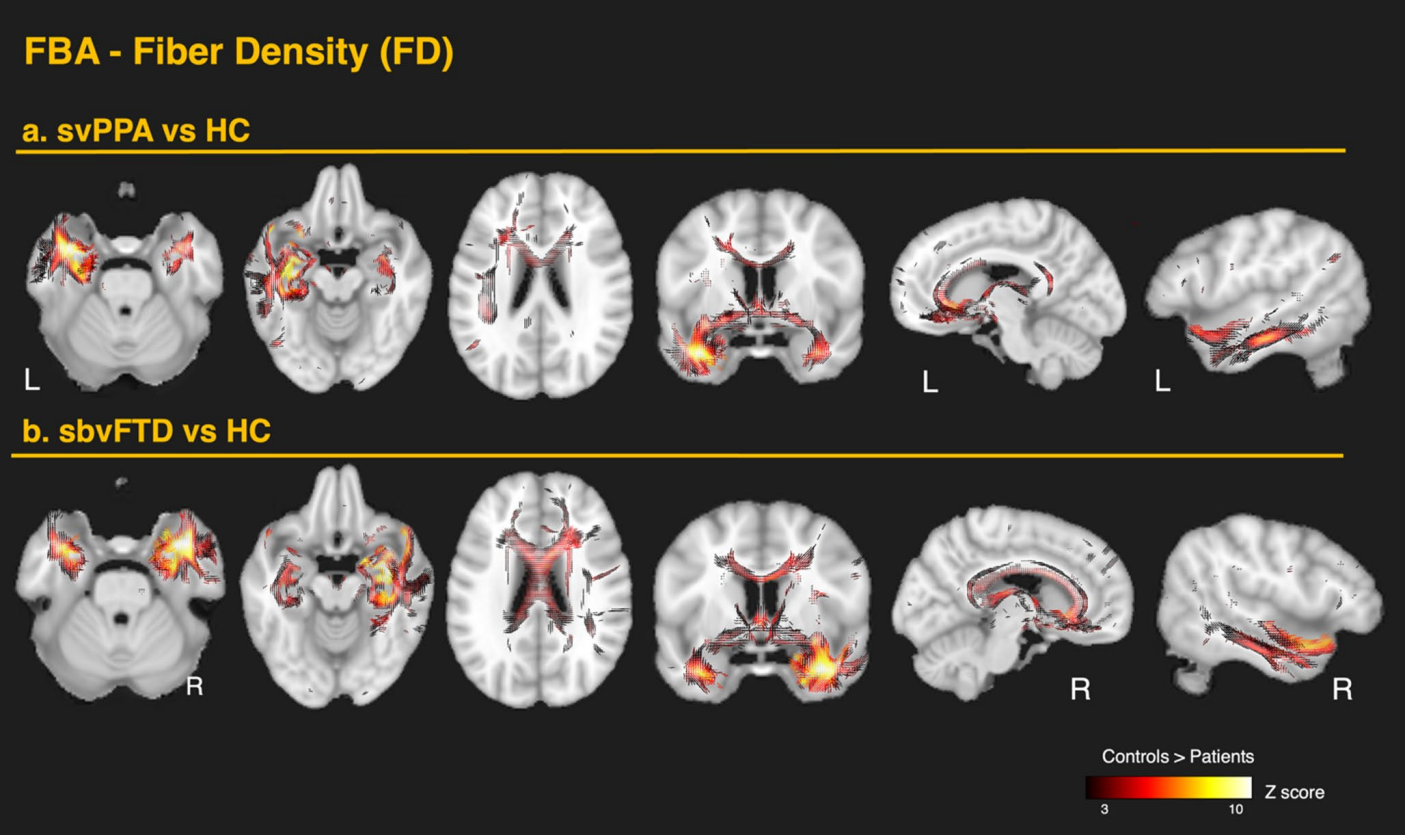
Fixel-based analysis of fiber density loss in the two anterior temporal variants. Whole-brain fixel-wise contrasts of fiber density (FD) are shown on the MNI template, displaying fixels that survived family-wise error (FWE) correction at *p* < 0.05. **a** svPPA vs. controls. Pronounced left-lateralized reductions involve the uncinate (UF), inferior longitudinal (ILF) and inferior fronto-occipital fasciculi (IFOF, including orbitofrontal and fusiform projections), temporal projections of the arcuate fasciculus (AF), and the parahippocampal cingulum. Additional losses are noted in the anterior cingulate, in the anterior commissure, tapetum, splenium, and fornix. **b** sbvFTD vs. controls. A largely right-hemispheric mirror pattern emerges, but with more extensive bilateral ventral involvement. FD loss affects the ILF, UF, IFOF, temporal AF, parahippocampal cingulum. Additional losses are noted in the anterior cingulate, in the anterior commissure, tapetum, splenium, and fornix. Significant reductions extend throughout the corpus callosum (genu, body, and splenium)

In the svPPA group, FBA revealed significant reductions of FD, FC, and FDC in the left ILF, UF, IFOF (including projections to the orbitofrontal areas and around the fusiform), in the temporal projections of the AF, in the anterior cingulate, and parahippocampal gyrus. The most prominent effects were observed in the affected hemisphere, with additional involvement of contralateral ventral structures. Changes were also evident in the anterior corona radiata and within the internal and external capsules. FDC reductions largely corresponded to overlapping FD and FC changes. Significant alterations were also observed in the anterior commissure, tapetum, cingulum bundle, and fornix – pathways that are less easily detected in skeleton-based TBSS analysis. A summary of the FBA findings for svPPA is shown in Fig. 2B. Overall, FC exhibited greater impairment than FD, with the strongest effects along WM tracts connecting the ATL to mesial temporal structures such as the amygdala and parahippocampal gyrus. Detailed results are presented in Figs. 4A (FD) and 5A (FC). All FBA metrics showed significant decreases in the subgenual cingulum, while FC and FDC reductions extended from the anterior to the midcingulate area. Conversely, significant reductions in the anterior CC were observed only in FD and FDC, with no changes in FC - potentially reflecting distinct or secondary pathological processes beyond focal atrophy.

#### Differences in SbvFTD versus controls

In the sbvFTD group, FBA demonstrated a mirrored pattern of WM alterations relative to svPPA, with more extensive bilateral ventral involvement. A summary of the FBA findings for sbvFTD is shown in Fig. 3B. The most pronounced reductions in FD occurred bilaterally in the ILF and UF. Significant reductions were also found in the IFOF, temporal projections of the AF, cingulate projections to the parahippocampal gyrus, anterior commissure, tapetum, fornix, anterior corona radiata, and both internal and external capsules. In contrast to svPPA, the sbvFTD group showed significant changes throughout the entire CC. As in svPPA, FC reductions were more extensive than FD reductions (Fig. 3B). These changes extended dorsally from the temporal pole of the affected hemisphere to the inferior temporal gyrus, fusiform area, and the inferior parietal area, including the angular gyrus. Detailed results are shown in Figs. 4B (FD) and 5B (FC). As in svPPA, FC and FDC reductions, but not FD changes, were detected bilaterally in the anterior cingulum, while FD and FDC reductions, but not FC, were observed throughout the entire CC, including the posterior segments.

.

**Fig. 5:**
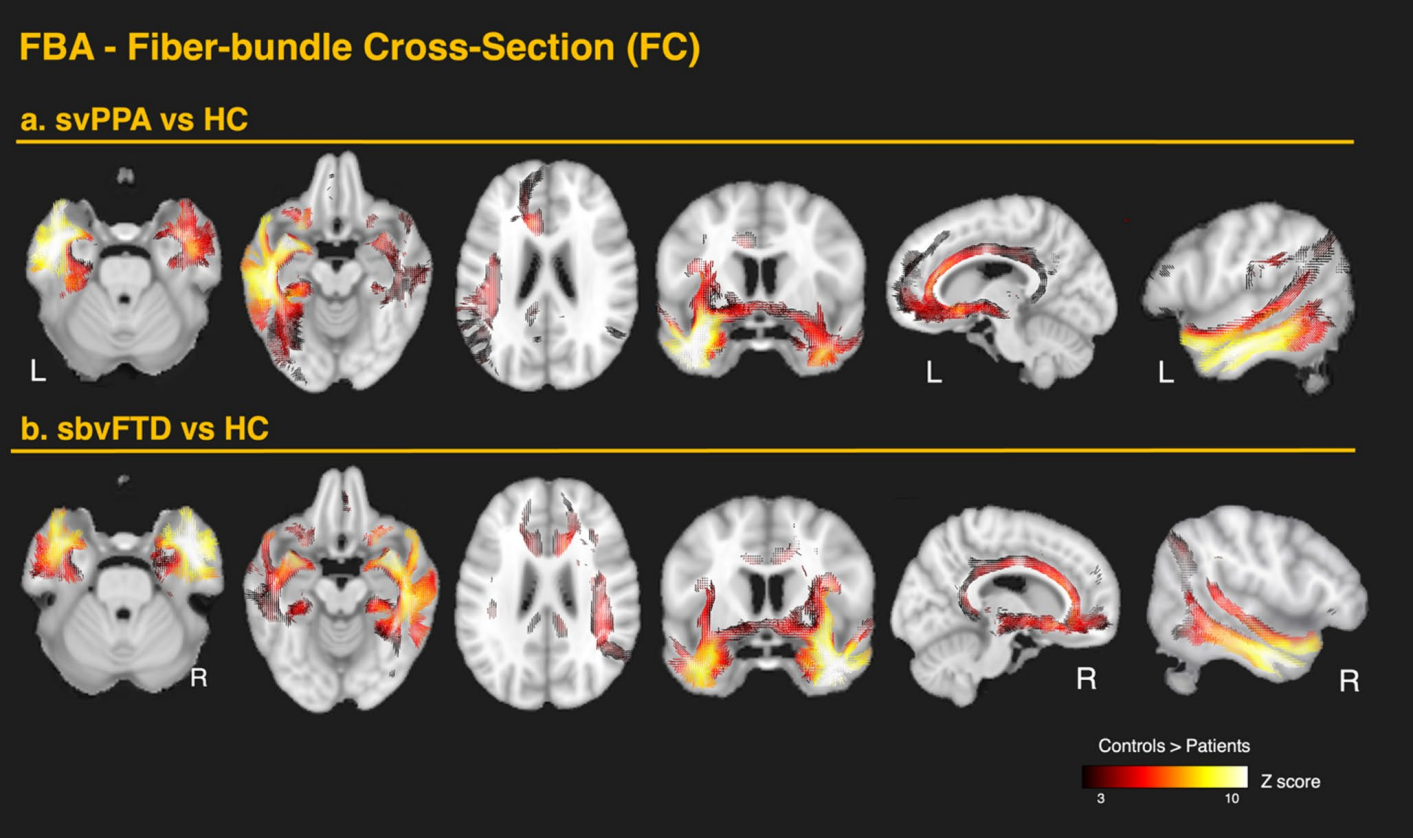
Fixel-based analysis of fiber-bundle cross-section in the two anterior-temporal variants. Whole-brain fixel-wise contrasts of fiber-bundle cross-section (FC) are shown on the MNI template, displaying fixels that survived family-wise error (FWE) correction at *p* < 0.05. **a** svPPA vs. controls. Marked left-lateralized bundle narrowing spans ventral association tracts (UF, ILF, IFOF), fornix, parahippocampal cingulum, anterior commissure, and tapetal callosal fibers, with extension into the long segment of the AF (parietal projections). FC reductions also encompass the subgenual and mid-cingulate portions of the cingulum, the anterior commissure, tapetum, splenium, and fornix. **b** sbvFTD vs. controls. A right-lateralized yet bilaterally extensive pattern appears. FC reduction mirrors ventral temporal pathways and extends dorsally from the temporal pole through inferior temporal cortex to the inferior parietal/angular gyrus. FC reductions are also evident in the anterior–mid cingulum, anterior commissure, tapetum, splenium, and fornix

## Discussion

This study provides novel insights into WM degeneration in SD by applying both TBSS-DTI and FBA to multi-shell diffusion MRI data. As hypothesized, the fiber-specific modeling enabled by FBA identified subtle alterations and additional WM pathways beyond those captured by TBSS, particularly in interhemispheric ATL connections such as the anterior commissure. These results underscore the value of fiber-specific approaches for detecting fine-scale network disruptions underlying neurodegenerative diseases. In the following sections, we discuss the technical advantages of FBA and the novel anatomical insights it provides regarding ATL connectivity in semantic dementia.

### Comparison of TBSS versus FBA: advantages and challenges

Diffusion MRI is currently the only in vivo technique capable of characterizing WM fiber architecture, making it a crucial tool for studying connectivity in neurodegenerative diseases. TBSS has been widely used due to its robustness in aligning FA maps across subjects and its ability to reduce partial volume effects by restricting analyses to a WM skeleton. This approach minimizes misregistration errors and allows for reliable voxel-wise statistical comparisons of DTI metrics such as FA and MD (Smith et al. 2006). However, TBSS has well-documented limitations, including its inability to resolve crossing fibers —ubiquitous throughout the brain—and its reliance on projecting diffusion data onto a group-averaged skeleton, which can lead to signal loss in peripheral or anatomically complex regions (Bach et al. 2014; Zalesky 2011). FBA represents a significant methodological advancement by enabling fiber-specific quantification within each voxel (Raffelt et al. 2017). Unlike TBSS, which averages scalar values across all fiber populations within a voxel, FBA disentangles individual fiber populations (fixels), allowing for the estimation of fiber-specific metrics such as FD, FC, and their combined product FDC. These metrics improve sensitivity to both microstructural degeneration and macrostructural atrophy, particularly in regions with complex fiber architecture. In the context of neurodegeneration, FBA has been successfully applied to detect tract-specific changes not captured by conventional DTI approaches (Dewenter et al. 2023; Mito et al. 2018; Oh et al. 2021; Rau et al. 2019; Savard et al. 2022). Notably, Savard et al. (2022) demonstrated widespread reductions in FC and FD across all FTD variants. In svPPA specifically, they reported left-lateralized FC reductions in the ILF—a key ventral pathway—highlighting FBA’s ability to resolve variant-specific degeneration with greater anatomical specificity than standard diffusion approaches. Their findings also support the notion that FC may be more sensitive than FD in capturing the spatial extent of white matter damage, a pattern consistent with our results. In our study, we further extended these findings by examining finer grained and under-characterized pathways, including the anterior commissure and projections to mesial temporal and limbic structures. These results illustrate the additional anatomical precision afforded by FBA, particularly in detecting degeneration of small or juxtacortical tracts that may be overlooked by skeleton-based approaches such as TBSS. However, it should be noted that FC and FDC partly reflect macroscopic volumetric deformation derived from the registration process (via the Jacobian determinant) in addition to microstructural diffusion signals. This overlap with morphometric changes underscores the need for cautious interpretation of these metrics, especially in regions of pronounced atrophy.

Both TBSS and FBA identified largely overlapping major pathways, their spatial coverage and interpretative scope differ. TBSS is constrained to the white-matter skeleton and provides voxel-wise measures of diffusion tensor properties, whereas FBA operates on the full fixel-wise white-matter model, quantifying both fiber-specific microstructure and macrostructural deformation. Accordingly, the two methods are not directly comparable; rather, they offer complementary perspectives on white-matter degeneration. In this study, we emphasize their convergence in key tracts and the added anatomical detail provided by FBA.

### Novel insights into interhemispheric ATL connectivity

Our results highlight the anterior commissure and the tapetum as critical conduits for cross-hemispheric spread of pathology in SD. The AC links the anterior-inferior temporal cortices and, as Fletcher and Warren (2011) argued, may provide the structural route through which pathology propagates between temporal lobes in network-level disconnection syndromes. Technical limitations have left only sparse empirical evidence of AC degeneration in SD (Moon et al. 2008). Using FBA, we detected convergent reductions in both FC and FD within the anterior commissure, most pronounced in sbvFTD. This parallels the broader bilateral gray matter involvement in sbvFTD and supports the commissure’s role in inter-hemispheric disease spread. Future studies correlating FBA-derived metrics with behavioral and cognitive profiles will further clarify the functional significance of anterior commissure degeneration in SD. The tapetum, the CC segment that joins the medial temporal lobes, also emerged as a key structure. FBA revealed reduced FD with relatively preserved FC, consistent with microstructural injury that precedes overt tract atrophy. Longitudinal diffusion MRI work in svPPA has already documented progressive tapetal degeneration (Elahi et al. 2017); our findings indicate that such alterations are detectable at earlier disease stages. Because these microstructural changes precede macroscopic volume loss, tapetal FD could represent an early biomarker for tracking progression (Downey et al. 2015; Tu et al. 2016). Although semantic dementia is usually driven by FTLD-TDP type C pathology, how pathological TDP-43 crosses from one hemisphere to the other remains uncertain. Prion-like, trans-synaptic spread is well established for aggregated tau, yet whether TDP-43 follows the same route or employs a different mechanism is still under debate, despite emerging evidence that hints at prion-like behavior (Keszycki et al. 2022). Commissural pathways—such as the anterior commissure, tapetum, and broader anterior CC—are anatomically plausible conduits, but direct proof of TDP-43 trafficking along these tracts is lacking. Combining ultra-high-field diffusion imaging with post-mortem tract-tracing and biochemical studies will be crucial to test this hypothesis and clarify how interhemispheric connectivity influences clinical phenotype and progression in SD.

### Greater definition of mesial-temporal connectivity in semantic dementia

Our results reveal damage to the limbic/temporo-mesial pathways—the UF cingulum bundle, and fornix—that interlink the hippocampus, amygdala, anterior thalamus, and orbitofrontal cortex (Catani et al. 2013; Jumah and Dossani 2024). Although gray matter atrophy of the amygdala and hippocampus in SD is well-documented (Boxer et al. 2003; Brambati et al. 2009; Chan et al. 2001; Collins et al. 2017; Hodges and Patterson 2007, p. 2; Marshall et al. 2018; Mummery et al. 2000; Rosen et al. 2002), limitations of conventional diffusion MRI have hampered investigation of their white-matter connections. FBA enables detection of microstructural loss in these small tracts—changes largely missed by DTI/TBSS—thereby providing evidence of their involvement in SD. The UF, running from the anterior temporal lobe to orbitofrontal cortex, integrates visceral–emotional cues with semantic memory and decision-making; its degeneration aligns with the combined semantic and socio-emotional deficits observed in SD (Agosta et al. 2010; Catani et al. 2013). The cingulum bundle links medial temporal, limbic, and medial frontal regions; our finding of reduced fiber density, together with earlier reports of selective von Economo neuron loss in the anterior cingulate (Lam et al. 2014; Tan et al. 2014), helps account for the behavioral and affective changes typical of SD (Landin-Romero et al. 2016). The fornix, the principal efferent of the hippocampus, showed lowered fiber density in our cohort, echoing prior reports that disruption of the fornix predicts memory and emotional impairments in SD and underscoring FBA’s value for detecting limbic micro-degeneration (Elahi et al. 2017; Hodgetts et al. 2017; Modi et al. 2013).

Taken together, these limbic alterations demonstrate that SD pathology extends well beyond the anterior temporal cortex, undermining a broader network essential for memory, emotion, and behavioral regulation. Future ultra-high-field diffusion imaging studies, combined with tract-specific functional and behavioral measures, will be crucial for clarifying how limbic disconnection shapes disease trajectory and for identifying targets for neuromodulatory or behavioral interventions aimed at preserving residual connectivity.

### Major white matter bundle involvement in semantic dementia

Previous diffusion tensor studies have consistently demonstrated degeneration of ATL projection tracts in SD – most consistently the ILF, UF, IFOF, and AF (Acosta-Cabronero et al. 2011; Agosta et al. 2010, 2015; Galantucci et al. 2011; Mahoney et al. 2013; Mandelli et al. 2014; Tu et al. 2016; Whitwell et al. 2010). Our combined TBSS and FBA approaches corroborate these findings, with the most pronounced abnormalities in the hemisphere showing greater gray-matter loss. As in earlier work, RD proved more sensitive than FA for detecting these changes (Acosta-Cabronero et al. 2011; Agosta et al. 2010; Mahoney et al. 2013; Schwindt et al. 2013). Functionally, the ILF links the ATL to occipital-temporal visual regions that support object recognition; its disruption correlates with naming deficits, impaired semantic access and difficulties recognizing faces and emotions (Catani and Thiebaut de Schotten 2008; Herbet et al. 2018). The IFOF supports multimodal semantic control; left-hemisphere damage is associated with verbal semantic deficits, whereas right-hemisphere damage is linked to socio-emotional impairments (Gonzalez Alam et al. 2024; de Zubicaray et al. 2011). The UF connects the ATL to orbitofrontal cortex, bridging semantic and limbic networks; left-UF injury is linked to language-semantic deficits, while right-UF injury more often affects socio-emotional behavior (Oishi et al. 2015; Toller et al. 2022; Von Der Heide et al. 2013). Finally, AF abnormalities extend into inferior-parietal territory, indicating that white-matter damage may precede overt cortical thinning in these regions and highlighting the need to monitor dorsal as well as ventral pathways longitudinally. Taken together, degeneration of these tracts helps explain the heterogeneous clinical presentations of semantic dementia. Mapping their differential vulnerability provides a framework for tracking disease progression and for targeting future therapeutic or neuromodulatory interventions to specific white-matter pathways.

### Limitations

Despite its strengths, this study has some limitations. First, the sample size was modest because we restricted inclusion to patients in the early disease stage who also had multi-shell diffusion imaging data. While this strategy enabled precise characterization of early pathology, it inevitably reduced statistical power and may limit the generalizability of our findings. Nonetheless, our sample size is comparable to or larger than most prior diffusion studies of semantic dementia, which typically include between 5 and 26 patients per group (e.g., Agosta et al. 2010; Galantucci et al. 2011; Whitwell et al. 2010; Savard et al. 2022). The use of high-angular-resolution, multi-shell diffusion imaging further enhances statistical sensitivity despite the modest sample size inherent to this rare disease population. Second, we did not include direct correlations between WM changes and clinical or behavioral measures as the current study was designed to delineate the anatomical patterns of unilateral and bilateral disruptions in WM connectivity, rather than to link these imaging findings directly to clinical or behavioral outcomes. Future studies in larger cohorts will integrate multimodal imaging and behavioral data to clarify how these structural changes relate to symptom profiles. Third, registration or normalization errors during FBA processing could bias fiber-cross-section estimates—especially in regions of pronounced atrophy, where accurately aligning white-matter structures is intrinsically difficult. Nevertheless, the mirrored results obtained in left- and right-predominant ATL groups argue against major misalignment artifacts. Moreover, diffusion-derived metrics such as FD, FC, and FDC are indirect proxies of WM integrity and may also be influenced by factors beyond axonal loss, including myelin changes, iron accumulation, or signal dropout. Future multimodal and postmortem validation studies will be crucial to confirm the biological specificity of these measures. Finally, in severely atrophic areas it remains challenging to disentangle gray- and WM damage, calling for cautious interpretation of tract-based metrics. Collectively, these limitations highlight the need for larger cohorts, further refinements to FBA registration pipelines, and rigorous quality-control procedures in future work.

### Conclusion and future directions

Advanced diffusion imaging adds crucial anatomical detail to our understanding of WM involvement in SD. Traditional skeleton-based methods such as TBSS capture the broad pattern of degeneration but may miss many subtle changes due to their limited ability to resolve crossing fibers or identify very small tracts. FBA overcomes these limitations, detecting both microstructural loss (reduced fiber density) and macrostructural atrophy (reduced fiber cross-section) in pathways that are difficult to characterize with conventional techniques—most notably the anterior commissure and mesial-temporal projections to the amygdala and hippocampus—while also refining our view of established ATL-connected tracts. Nonetheless, diffusion-based metrics should be interpreted with caution, as they represent indirect proxies of microstructural integrity and may be affected by other biophysical processes. Future multimodal and histopathological validation studies will be essential to confirm the biological specificity of these findings. Larger, longitudinal cohorts are now needed to test whether early FBA abnormalities precede—or merely accompany—the spread of cortical pathology and whether they predict distinct clinical phenotypes. Such work will determine the prognostic value of tract-specific metrics and help explain the heterogeneity of semantic dementia.

Finally, high-resolution diffusion metrics could guide therapeutic strategies. By pinpointing residual connectivity, they may inform the placement or targeting of neuromodulation approaches such as transcranial magnetic or direct-current stimulation and aid in monitoring treatment response. Integrating sophisticated imaging with personalized interventions therefore holds considerable promise for improving clinical outcomes and for clarifying the neurobiological mechanisms that drive this disease.

## Supplementary Information

Below is the link to the electronic supplementary material.

Supplementary Figure 1. Population-averaged tractography atlas used for anatomical labelling. Axial, coronal, and sagittal views of the HCP-1065 population-averaged tractography atlas are shown on the MNI-152 template. Color-coded probability maps depict the major association, commissural, and limbic bundles analyzed in the present study: long segment of the arcuate fasciculus (AF, orange), posterior AF (yellow), superior longitudinal fasciculus III (SLF III, light blue), cingulum bundle (dark blue), inferior fronto-occipital fasciculus (IFOF, grey), inferior longitudinal fasciculus (ILF, green), uncinate fasciculus (UF, light green), anterior commissure (AC, white), corpus callosum (CC, beige), and fornix (red). These atlas masks were used to guide region-of-interest definition and to aid interpretation of fixel-based results shown in Figs. 4–5

## Data Availability

Upon publication, data from this study will be made available through an access-controlled repository within the FAIR Alzheimer’s Disease Data Initiative AD Workbench (https://fair.addi.ad-datainitiative.org). Requests for access can be submitted through the UCSF-MAC Resource Portal (Request form: http://memory.ucsf.edu/resources/data). Access will be granted following UCSF-regulated procedures in accordance with ethical guidelines for the reuse of sensitive data. Researchers seeking access must submit a Material Transfer Agreement, which is available at: https://icd.ucsf.edu/material-transfer-and-data-agreements.

## Abbreviations

WM: White matter
dMRI: Diffusion magnetic resonance imaging
DTI: Diffusion-tensor imaging
TBSS: Tract-based spatial statistics
FBA: Fixel-based analysis
FWE: Family-wise error
FA: Fractional anisotropy
MD: Mean diffusivity
AD: Axial diffusivity
RD: Radial diffusivity
FD: Fiber density
FC: Fiber-bundle cross-section
FDC: Fiber density and cross-section
SD: Semantic dementia
svPPA: Semantic variant primary progressive aphasia
sbvFTD: Semantic behavioral fronto-temporal dementia
MMSE: Mini-mental state examination
CDR: Clinical dementia rating
ATL: Anterior temporal lobe
ILF: Inferior longitudinal fasciculus
UF: The uncinate fasciculus
IFOF: Inferior frontal occipital fasciculus
AF: Arcuate fasciculus
CC: Corpus callosum
MPRAGE: Magnetization-prepared rapid acquisition with gradient echo acquisition
TIV: Total intracranial volume
