# Supplementary figures and images for: Fixel-based analysis reveals detailed white matter changes in semantic dementia
Source: Brain Struct Funct. 2026 Jan 7;231(1):9. doi: 10.1007/s00429-025-03064-7 (PMC12779712; doi:10.1007/s00429-025-03064-7)

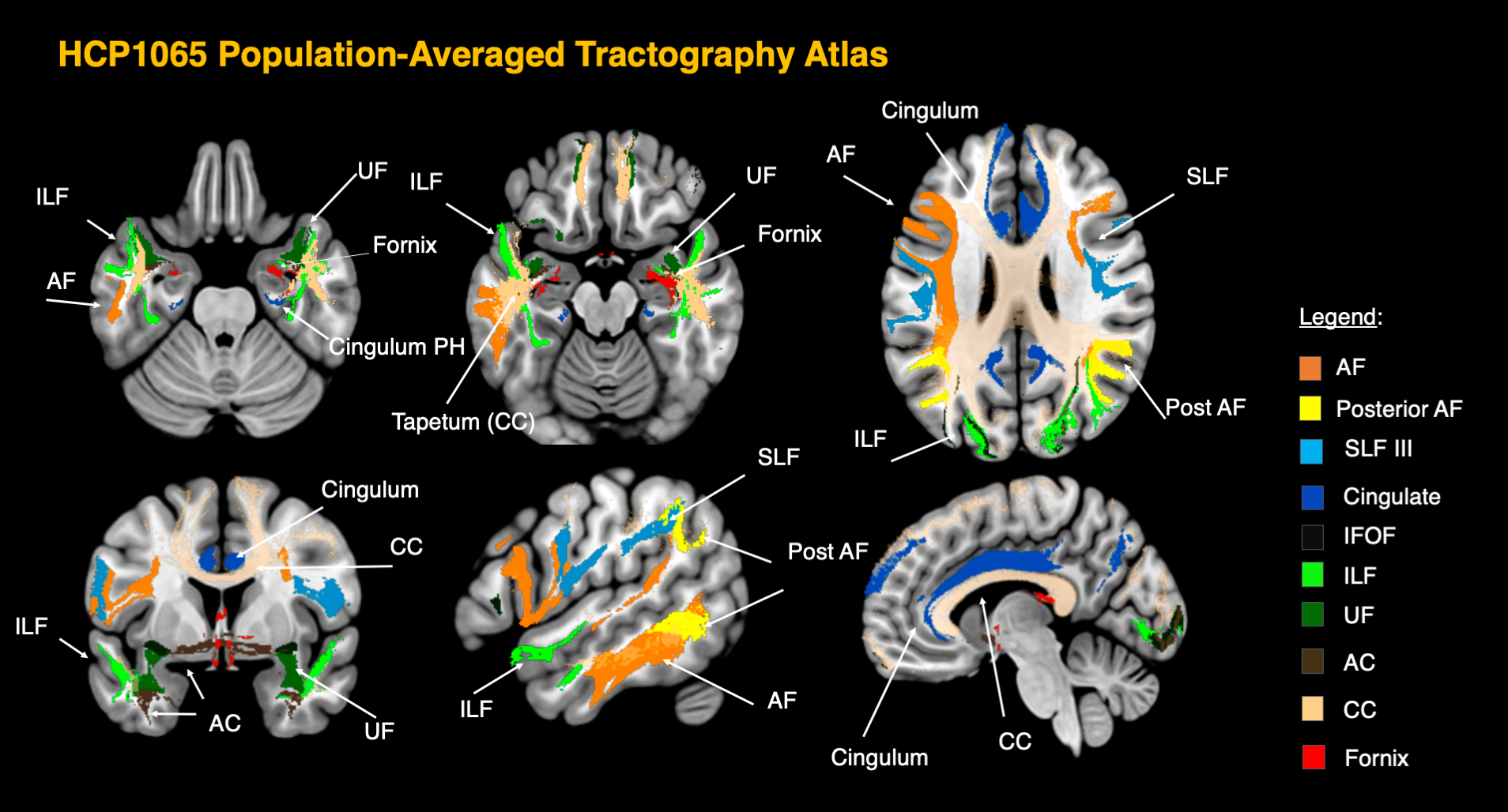

Supplement: Supplementary file 1 — Supplementary Figure 1. Population-averaged tractography atlas used for anatomical labelling. Axial, coronal, and sagittal views of the HCP-1065 population-averaged tractography atlas are shown on the MNI-152 template. Color-coded probability maps depict the major association, commissural, and limbic bundles analyzed in the present study: long segment of the arcuate fasciculus (AF, orange), posterior AF (yellow), superior longitudinal fasciculus III (SLF III, light blue), cingulum bundle (dark blue), inferior fronto-occipital fasciculus (IFOF, grey), inferior longitudinal fasciculus (ILF, green), uncinate fasciculus (UF, light green), anterior commissure (AC, white), corpus callosum (CC, beige), and fornix (red). These atlas masks were used to guide region-of-interest definition and to aid interpretation of fixel-based results shown in Figs. 4–5 [file 429_2025_3064_MOESM1_ESM.tiff]
